# Editorial: Health and welfare of rabbits (on a farm, in the laboratory or as a pet)

**DOI:** 10.3389/fvets.2024.1522420

**Published:** 2024-11-19

**Authors:** Michaela Fels, Livia Benato, Alexandra Whittaker

**Affiliations:** ^1^Institute for Animal Hygiene, Animal Welfare and Farm Animal Behaviour, University of Veterinary Medicine Hannover, Foundation, Hannover, Germany; ^2^University of Bern, ESF- Experimental Surgery Facility Inselspital, Julie-von-Jenner Haus, Bern, Switzerland; ^3^School of Animal and Veterinary Sciences, The University of Adelaide, Adelaide, SA, Australia

**Keywords:** animal welfare, rabbit farming, rabbit health, does, antibiotic resistance

Rabbits are kept by humans in a variety of ways and for very different purposes. They are not only popular pets but are also used for meat production and kept in cages on farms. Moreover, rabbits are kept as laboratory animals for scientific purposes for research on human and veterinary medicine and for the purposes of safety testing of consumer products. In all of these areas of use, husbandry practices, disease status, and treatment regimens differ. Consequently, research applying to one area may not easily translate to other areas of use, and many aspects of rabbits' health and welfare still require further research to improve housing systems and veterinary care. The articles published in this Research Topic focused primarily on meat-producing rabbits. The rabbit farming sector currently seems to be receiving significant research attention, which is reflected in the articles submitted for this Research Topic. The authors shared new scientific knowledge aiming to improve the lives of rabbits and prevent or counteract disease and stress in their housing environments. The collection of studies presented in this Research Topic deals with digestive impairments in rabbits, the risk of antibiotic resistance, and group housing of breeding does. All of these topics also have relevance to the keeping of rabbits as pets as well as laboratory animals. Thus, the three papers add a valuable contribution to the current literature that can be used for all areas of rabbit housing.

Digestive problems are very common in rabbits and often lead to high animal losses, especially in fattening rabbits (1). The article by van der Sluis et al. highlights the possible causes of indigestion in rabbits and identifies two pathways: the “overload” pathway and the “chymus jam” pathway. The “overload” pathway is caused by an excess of easily fermentable substrates in the feed, which reach the cecum incompletely digested. Normally, proteins and starch are mainly digested in the small intestine. If starch and proteins increasingly enter the cecum, the digestive processes that take place there lead to a change in the cecum pH, with a negative influence on the microorganisms located there. The other pathway (“chymus jam”) is caused by a reduced motility of the hindgut resulting from stress or a too-limited fiber supply. Ultimately, overfilling of the cecum and proximal colon occurs, thus resulting in a “chymus jam” and increased cecal retention times. This in turn leads to a negative influence on the microbial flora of the cecum. The authors explain that they wanted to shed light on non-specific digestive disorders in rabbits. This primarily, but not exclusively, relates to rabbits raised for meat production. Rabbit digestive tract mechanisms are similar whether animals are reared for meat production, used in labs, or kept as pets, and thus learnings from this paper apply to all. According to the authors, their findings could be a starting point for further research into feeding-related impairments of digestion in rabbits, with the aim of improving the health and wellbeing of rabbits in the long term.

Another important issue, which is highly relevant in the keeping of rabbits for meat production, is the growing concern of antibiotic resistance to various bacterial pathogens. In a study by Sun et al., the antibiotic resistance spectrums of *Escherichia coli* (*E. coli*) and *Enterococcus* spp. strains against commonly used antimicrobials from commercial meat-rabbit farms in Chengdu City, Southwest China was investigated. A total of 75 *E. coli* and 210 *Enterococcus* spp. strains were recovered from fecal samples of healthy meat-rabbits. On the two rabbit farms, diverse resistance phenotypes against seven commonly used antimicrobials were found. The tested antimicrobials were ampicillin (AMP), amoxicillin-clavulanic acid (A/C), doxycycline (DOX), enrofloxacin (ENR), florfenicol (FFC), gentamicin (GEN), and polymycin B (PMB). The results showed that the DOX-based resistance phenotypes for *E. coli* and *Enterococcus* spp. strains were predominant. The authors conclude that the prevalence of antimicrobial resistance in healthy rabbits supports the assumption that the use of therapeutic antimicrobials in rabbit farming may promote antibiotic resistance transmission among indicator bacteria. The authors recommended a periodic surveillance of antibiotic resistance in geographic locations and supervisory measures for rational antibiotic use. This would be a good measure in terms of animal health (not only farm animals) and human public health, and thus an important step in the spirit of One Health.

The third paper in this collection deals with part-time group housing of does and their litters on a commercial rabbit farm. In rabbit farming, breeding does are usually single housed after they have reached maturity. Single housing is supposed to prevent the does from injuries caused by aggressive interactions and to ensure reproductive success (2). However, single housing is contrary to the natural behavior of rabbits and could be a significant contributor to impaired welfare. Therefore, more studies are needed to find out how group housing of does can be realized, while avoiding aggressive interactions that lead to severe injuries. In the study by Van Damme et al., the effects of cage enrichment on aggression in part-time group-housed female breeding rabbits were investigated. In part-time group housing, the does are single-housed for kindling and regrouped after new insemination, which is usually followed by aggressive interactions. Aggressive behavior in rabbits, such as fighting or chasing, is related to the establishment and upholding of a social hierarchy within groups and cannot be completely avoided (3); however, it may be reduced by specific management strategies. Such a strategy could be the provision of manipulable material examined in the study by Van Damme et al.. The authors compared alfalfa blocks, wooden panels, both alfalfa and wooden panels, and no extra enrichment (control). In this study, does were housed in groups of four animals together with their litters from day 22 until day 32 post-partum. On all observation days, the number of injured does was higher in the control group than in the groups with alfalfa blocks and in the groups where both materials (alfalfa and wooden blocks) were provided. However, severe injuries still occurred in all groups. The authors conclude that the high prevalence of injured animals, even in the enriched multi-litter cages, indicates that more effective or additional strategies are needed to reduce the welfare problems in group housing systems for does. Much work still needs to be done in this field of research to find systems and strategies that would enable breeding females to be kept in groups and thus allow them to express their natural social behavior.

In summary, the studies published in this Research Topic have provided new insights into important issues related to rabbit husbandry. At the same time, the need for further research has been highlighted, as the topics relating to the wellbeing and health of rabbits are very complex and many things have not yet been scientifically clarified. Whether it is the intestinal health, the controlled use of antibiotics, or social behavior in group housing, there is a need for further research everywhere. This Research Topic is therefore intended to encourage continued intensive work on issues relating to the welfare of rabbits so that new ways can be found to improve their wellbeing.
